# Shape Analysis of the Elastic Deformation Region throughout the Axi-Symmetric Wire Drawing Process of ETP Grade Copper

**DOI:** 10.3390/ma14164713

**Published:** 2021-08-20

**Authors:** Paweł Strzępek, Andrzej Mamala, Małgorzata Zasadzińska, Grzegorz Kiesiewicz, Tadeusz Antoni Knych

**Affiliations:** Faculty of Non-Ferrous Metals, AGH University of Science and Technology, 30-059 Kraków, Poland; amamala@agh.edu.pl (A.M.); malgozas@agh.edu.pl (M.Z.); gk@agh.edu.pl (G.K.); tknych@agh.edu.pl (T.A.K.)

**Keywords:** wire drawing process, Cu-ETP, elastic deformation region, plastic deformation region, numerical and experimental analyses

## Abstract

The wire drawing process is commonly perceived as one of the best studied metal forming processes in almost every aspect; however, when considering elastic deformation, researchers usually focus on the uniaxial tensile forces after the material exits the drawing die and not the elastic deformation region before entering the drawing die, even though it may have a significant impact on the strength parameters and the nature of metal flow inside the drawing die. The aim of this research is to theoretically and experimentally identify the deformation in the elastic region and to further link the shape of this region and the values of stress occurring in it with the geometrical parameters of the drawing process and assess its impact on its strength parameters. In order to achieve the assumed goals, numerical analyses using the finite element method and experimental research on the drawing process in laboratory conditions were carried out using Vickers hardness tests and resistance strain gauges measuring deformation in stationary and non-stationary conditions. The obtained results indicate that the shape and the extent of the region of elastic deformations generated in the material before the plastic deformation region during the drawing process depends on the applied deformation coefficient and stationarity of the process.

## 1. Introduction

It is commonly accepted that the drawing process of round wires is one of the most well-known metal forming processes, both theoretically and technologically, not only due to its circular symmetricity and stationarity but also because many formulas allow for fairly accurate calculations of drawing forces (e.g., Sachs or Siebel equations and analyses), the coefficient of friction, residual stresses in the material, unit pressure values, etc. [1,2,3,4,5]. However, throughout the process of manufacturing mostly profiles but also round wires, there are a number of technological difficulties related to the obtaining of the desired mechanical properties, required dimensional tolerances, and satisfactory surface quality. Longitudinal or cross-sectional cracks, which often occur during the process, make the drawn material completely unsuitable for further processing. This phenomenon may transpire mainly due to the loss of metal plasticity, causing well-known drawing defects such as chevron cracks, bulging, or thinning, which are caused by improper technological conditions of the process (e.g., inaccurate deformation coefficient, die angle, coefficient of friction, or velocity field of plastic deformation region) [6,7,8]. Technological aspects of wire drawing failures were analyzed by Raskin et al. [9] based on 673 wire breaks, which happened in industrial conditions throughout the drawing process of pure copper with the use of a multi-wire drawing machine. They stated that inclusions are the main cause of 52% of wire breaks; however, it is worth noting that as much as 13% of material discontinuities among the tested samples were caused by the aforementioned defects such as chevron cracks, commonly known as central bursts, or cup and cone breaks.

**Figure 1:**
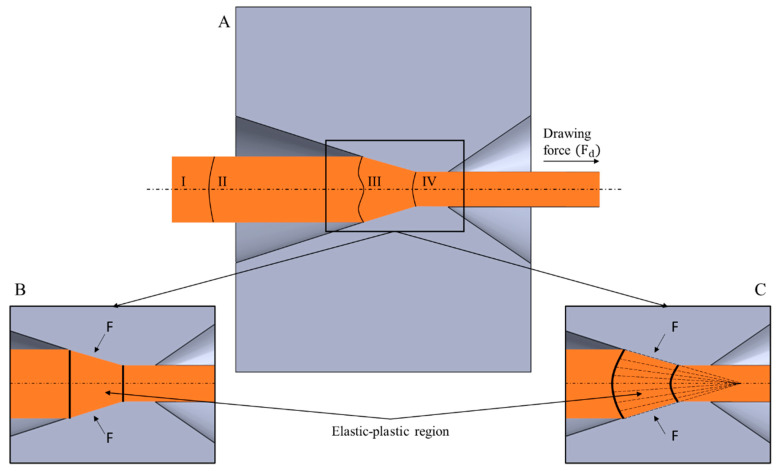
Deformation regions inside of the drawing die (**A**)—theoretical analyses of wire drawing process (classical approach): (**B**) the flat cross-section hypothesis (homogenous deformation); (**C**) the spherical boundary hypothesis (upper-bound approach); based on [10]. Reproduced with permission from Knych, Elastic and Plastic Strains in Drawn Circular Sections; Uczelniane Wydawnictwa Naukowo-Dydaktyczne: Kraków, Poland, 2001.

It is assumed that when an elastic–plastic body is subjected to a sufficient stress field, the plastic deformation region is generally accompanied by an elastic deformation region. When considering the wire drawing process, the location of the elastic–plastic region is usually situated inside of the die reduction angle, and to meet the criteria required for material transition to plastic state, it is necessary for it to be entirely proceeded by the elastic state. For the purposes of this analysis, four regions of strains throughout the length of the drawn material were distinguished and are presented in Figure 1A based on the descriptions made by Knych [10]: the unloaded region (I); the region of elastic deformation generated behind the elastic–plastic region (II); the elastic–plastic region (III); and the elastic region in which elasticity comes from the drawing force (IV).

Two parallel hypotheses concerning theoretical analysis of the wire drawing process may be considered regarding the classical literature on the subject, i.e., the flat cross-section hypothesis, which assumes that a selected set of points constituting a plane perpendicular to the drawing axis before entering the drawing die will remain at this plane throughout the entire process and after its completion (Figure 1B); and the spherical boundary hypothesis, which assumes a constant motion field of particles in the deformation region of the die, which means that during the plastic deformation, all points move towards the top of the cone formed by the drawing die (Figure 1C). Thus, the boundary of the elastic–plastic deformation region is expected to be a plane or a slice of the sphere. Such an assumption allowed for the use of analytical methods to solve problems existing inside of the drawing die.

Regarding more recent scholarly sources, however, attempts have been made to determine the elastic–plastic deformation boundary inside of the die reduction angle, e.g., using the method of characteristics [11], using the numerical analyses [12], or with the use of empirical studies such as performed by Dobrov [13] during the analysis of the shape of the deformation region from the side of the bearing length. The accessible data show that the shape of the elastic–plastic deformation region and, to some extent, the shape of the elastic deformation region related to it may be much more complex than is commonly assumed, and what is more, it may have a significant impact on the nature of the metal flow throughout the drawing process. It is worth noting that the length of the region in the analysis of [11] reaches a minimum value at the wire drawing axis, and as the deformation, being the result of the adopted process parameters, does not cover the entire material cross-section, it will lead to the forming of the above-described drawing defects, such as chevron cracks.

A wide range of scientific papers concerning the generally understood process of drawing of round wires were written in the 20th and 21st centuries, both in terms of the conventional drawing process and innovative methods, such as accumulated angular drawing (AAD), which tests the influence of the linearity of dies throughout the process; drawing with the use of ultrasound; drawing with an elevated temperature of the die [14]; or drawing with the use of high-density electric current pulses [15] or annealing by electropulsing during pure copper wire drawing, which allows the annealing time during the wire drawing process of pure copper to be shortened, and thus does not strengthen the final product [16]. Lack of strain hardening during the wire drawing process has also been observed after specific heat treatment of other materials [17]. Many researchers nowadays put more interest in the cryogenic wire drawing process as an alternative process that may increase the material’s mechanical properties, such as ultimate tensile strength, with the cost of higher drawing forces [3,18,19,20]. In their research paper, Massé et al. [21] attempted to re-evaluate the optimal drawing angle using finite element method analyses, which led them to conclusions that a lower drawing angle than considered to be optimal in the classic literature allows one to reduce the number of product defects with no significant increase in the drawing force; however, according to the authors, due to the influence of the friction conditions, it seems more advantageous to use a slightly higher drawing angle. Another thought-provoking academic work was conducted by Haddi et al. [22], who made an attempt to assess the influence of the drawing velocity and the temperature of the process resulting from the friction on the drawing force necessary to carry out the process. They stated that these values differ throughout the process, which is a result of the varying friction coefficient and the nature of metal flow, depending on the drawing velocity. Based on the obtained experimental results, a modification of the Avitzur model was proposed, which, in the opinion of the authors, will allow for the adjustment of the drawing parameters by minimizing the drawing stress of copper-based materials. Another study dealing the influence of die geometry on the drawing force based on numerical simulations was conducted by Sas-Boca et al. [23], who claimed that both die angle and bearing length have a significant influence on the calculated drawing force values. Results obtained in all these scholarly works show promising possibilities in terms of properties of the final product or decreased drawing force, which in effect would lower the energy expenditure and cause plausible changes in the plastic and elastic deformations of the material. These are, however, considered to be unconventional and difficult to implement in industrial conditions.

Most of the available wire drawing process scientific analyses are based on the rigid-plastic body model. Such simplification does not fully take into account the phenomena occurring in the material before entering the elastic–plastic deformation region inside of the drawing die, and the assumption that the material before entering the die reduction angle is stress-free is not true, as it is obvious that elastic–plastic deformations must be preceded by elastic deformations [24]. Among research works taking that into account, Martinez et al. [25,26] investigated the influence of the wire drawing parameters throughout the process, as well as die geometry on generated heat, friction, and plastic deformations. They confirmed that as a result of the material flowing in the direction of the die axis, the stresses changed from compressive to tensile. What is more, they also noticed that the stresses at the surface of the wire take higher values as the contact length of the material with the die reduction angle decreases, which results in later deformation heterogeneity. They also proved that as the drawing angle increases, the compressive stresses both at the surface and along the axis of the wire increase, and the material shows greater uniformity of the material flow in the radial direction. Additional studies proved that the reduction of the friction coefficient causes an increase in radial deformations. Similar observations have been made when the drawing angle has been changed. The authors of these two works [25,26] did not notice any influence of the die geometry on the axial deformations. Skołyszewski et al. [5], in their research work, investigated the influence of back tension throughout the wire drawing process of steel on recorded stress values and noted that despite the very short length of the region of elastic deformations, its values exceeded several times the value of the metal unit pressure on the wall of the drawing die, which is an extremely dangerous phenomenon during the wire drawing process. They observed, however, that the use of back tension lowered the recorded values at the boundary of the elastic and elastic–plastic deformation regions, which consequently led to lowering the values of unit pressure in the elastic deformation region. The authors in [27] analyzed the drawing force, stresses, and properties of the final product of the conventional wire drawing process of steel in comparison with the roller dies implemented in the process. The obtained results showed an increase in the drawing force and the die and material temperature throughout the unconventional process along with an increase in recorded stresses and deformation heterogeneity. The authors of this study did not record any changes in the properties of the final product; however, they noticed a significant improvement in the surface quality of the wire obtained with the roller die. Another analytical study in the area of various types of dies and their impact on the process was conducted by Zhang et al. [28] on aluminum alloy 7A09 with a conical die, single elliptical die, and twin elliptical die. They concluded through their numerical simulations and calculations that the lowest value of the drawing force should be expected when using the twin elliptical die. What is more, they also stated that the minimal values of stress should occur when using dies with an optimum drawing angle, with one additional condition that the angle should increase as the deformation coefficient increases. Some of the few empirical studies in recent years, conducted by Vega et al. [29] and Tintelecan et al. [30], proved that the drawing force in the process of copper wire drawing is influenced by the drawing angle, friction coefficient, and bearing length. The authors in [31,32,33,34] claim that there is a clear connection between the amount of oxygen and therefore copper oxides in the input material on the strength parameters of the materials, and thus the drawing forces, during the process. There is a noticeable modern trend towards lowering the occurring drawing forces, for instance by using various innovative lubricants such as graphite [35], PTFE [20], powdered soap [17], or MoS2 [3]. Various coating materials and lubricants have been investigated in [36] in terms of defects occurring on the surface of the wire after the metal forming process, and the authors proved that the smaller the size of the powder, the better the lubrication process. Additionally, they provided evidence that the larger size of the lubricant powder may cause delamination of the coating layer. When considering the surface of the metal wire, it has to be mentioned that microhardness, nanohardness, and Young’s modulus strongly depend on the state of the material surface and change upon contact, as has been proven both experimentally [37,38] and in numerical simulations [39]. In particular, electrical contact and the influence of the electric potential upon contact with other metal on a set of physical and mechanical properties were investigated throughout these research works, as the authors provided evidence that, for example, microhardness may decrease by as much as 8% when the electric potential of ~0.02 V is applied.

There are many scientific works concerning various metal forming processes and the elastic and elastic–plastic deformations occurring during the process, such as electroplasticity-assisted bending process [40], where the authors reduced the bending force and the elastic restoring force. Thermal elastic–plastic analysis was proposed by Kim et al. [41] during the friction stir welding process, where they claimed that the proposed parameters during the process would reduce the testing period and the cost of the manufacturing process and increase productivity of electric vehicle battery frames. The authors in [42] investigated the influence of various strain hardening models under cyclic loading on the elastic–plastic behavior of the material and provided data for further practical applications. These recent works, among many others, prove that elastic and elastic–plastic deformations are recently being given more consideration. Nonetheless, most of the modern research works concerning the wire drawing process and the forces, stresses, and deformations resulting from it are based on numerical and analytical simulations, which necessitate their verification in real conditions, thus proving the innovative approach of the research conducted in this work, which is focused on an empirical attempt to determine the length of the elastic deformation region in the material before entering the drawing die in the process of axi-symmetric wire drawing of ETP grade copper (electrolytic tough pitch). The research was carried out given that, according to the scholars and academics referenced in the introduction, the elastic deformation region may have an influence not only on the drawing force but also on the occurrence of drawing defects caused by heterogeneity of deformation on the cross-section of the final product.

## 2. Industrial Approach and Application

The focus of the article in on the fundamental research of a cognitive nature and not on application aspects. The conditions of the conducted experiment (velocity of the process and exogenous factors) vary from those in the drawing operations carried out on an industrial scale. Their selection was the key element necessary to isolate the tested parameters, such as the changes in the drawing force and elastic deformation of the material before entering the drawing die, which are normally concealed by changes in the temperature of the material.

Nevertheless, on the basis of the analysis of the conducted research, it is possible to propose a number of significant postulates important from a practical point of view and transfer them (after appropriate verification) to the industrial drawing process.

The observed increase in the drawing force in the non-stationary phase of the drawing process may be associated with the evolution (disappearance) of the elastic deformation region situated in front of the plastic deformation region. Therefore, it may be hypothesized that other process parameters (the presented study proved the influence of the size of the deformation coefficient λ) also have an impact on the shape of the elastic deformation region, and thus on the value of the drawing force in the stationary phase. After empirical verification of this hypothesis, it is possible to search for a new concept of minimizing the force parameters of the drawing process.

As is commonly known, in industrial drawing processes, the elastic back tension of a controlled value is often used in order to reduce the unit pressure of the material on the surface of the drawing die. At the same time, should the critical value be surpassed, it causes a significant increase in the drawing force and lowers the material’s deformability. This back tension is in synergy with the natural region of plastic deformation analyzed in this study. There is a prospective possibility that not only the value of the back tension, but also the elastic deformation region tested in the current work, affects the value and distribution of metal unit pressure on the drawing die’s surface, and thus the wear and tear of the tool or lubricants used. Moreover, the unit pressure and the coefficient of friction determine the temperature gradient across the material cross-section (temperature increase of the surface layer of the tribological origin), which to some extent determines the state of residual stress in the wire after the drawing process.

The studied phenomenon can also be analyzed from the point of view of defects occurring in drawn materials. The classical model of Avitzur’s central burst defects is based on a rigid-plastic body model, while in fact in the observed area of fractures, the material is probably in an elastic state. The elastic deformation region at the axis of the drawn material analyzed in this paper transforms into a new form and cracks appear, which depend not only on the kinematic incompatibility of the velocity field of the material particles, but also on the stress state. Recognition of this phenomenon gives hope for the development of a new mechanism for the emergence of central burst defects, and maybe even for the development of new technological recommendations, making it possible to eliminate the defects in question.

## 3. Materials and Methods

In order to empirically determine the length of the elastic deformation region generated behind the die in the axi-symmetric wire drawing process of copper, the authors of this paper conducted laboratory tests with as low as possible drawing velocity. Copper wire rod of ETP grade and chemical composition, as specified in Table 1, was chosen as an input material. Wire rod with a diameter of 20 mm was subjected to a prior wire drawing process on a laboratory draw bench machine in straight sections down to 11.55 mm and 10.5 diameter in 6 and 7 draws, respectively, without intermediate annealing, which assured the hard state of the input material and allowed the influence of strain hardening on the recorded values (approximation to the elastic-ideally plastic body model of the material) to be avoided. The first tests were conducted in order to determine the mechanical properties (ultimate tensile strength, yield strength, Young’s modulus, Poisson’s ratio, Vickers hardness) of the input material with a diameter of 11.55 mm using the uniaxial static tensile test, ultrasonic method, and Vickers hardness test.

The starting point of the conducted research were finite element method (FEM) simulations of the wire drawing process. The strain rates and stresses present at the wire cross-section were analyzed in order to identify elastic–plastic and elastic deformation regions, which functioned as a reference for empirical research conducted in real conditions.

**Figure 2:**
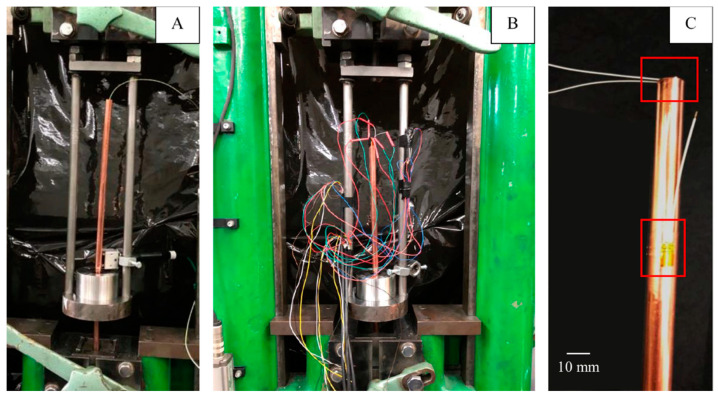
Test stands during the wire drawing process: (**A**) temperature verification with visible thermocouples; (**B**) elastic deformation analysis with visible strain gauges; (**C**) close-up of the sample with marked strain gauges (top—radial, bottom—axial in stationary conditions).

The most important part of the research was the experimental determination of the length of the elastic deformation region and the values of strains occurring in it in order to ensure that the authors carried out wire drawing tests at a very low drawing velocity (measured drawing velocity of the material after exiting the drawing die about 0.1 mm/s) on a testing machine (TIRA GMBH, Schalkau, Germany) with a maximum tensile force possible to be applied equal to 50 kN registered by an independent measuring system fixed with the jaws. The assembly was rigged with mechanical gears. The strain was measured using resistance strain gauges (HBM, Darmstadt, Germany) placed on the samples, as shown in Figure 2C, with a specially prepared test stand (Figure 2B), and the values were registered with Spider 8 measuring equipment (HBM, Darmstadt, Germany) and Catman software. This should have partially compensated for the temperature increase caused by the material friction against the die; however, in real conditions it is not possible to completely eliminate temperature increases during plastic working processes, so these values and their influence on the recorded strain values were analyzed using thermocouples, placed as shown in Figure 2A, and a thermal imaging camera (Optris, GmbH, Berlin, Germany).

Traditionally, the wire drawing process is considered to be stationary, and the macroscopic effect of this stationarity is the constant drawing force necessary to conduct this process. The authors made an assumption that if an elastic deformation region is being generated in the material before entering the die reduction angle, which in any way affects the strength parameters of the process, then from the moment when the material dimensions before the drawing die are smaller than the dimensions of the elastic deformation region, it is expected to have an effect on the drawing force. In order to verify this hypothesis, an analysis of the drawing process was carried out using a drawing die, with a diameter of 10 mm, using two various process parameters, i.e., with the deformation coefficient λ = 1.33 (input material with a diameter of 11.55 mm) and λ = 1.1 after an additional draw of the input material to diameter of 10.5 mm prior to strain gauge measurements, which would allow us to determine the influence of the applied cross-section reduction on the length of the elastic deformations region generated before entering the die reduction angle. An initial estimation of the length of the elastic deformation region was based on the observed increases in the recorded drawing force at the end of the process. More complex analysis of the length of the elastic deformation region was carried out using precise strain gauge measurements. In each case, measurements were made in stationary conditions, i.e., when the strain gauge was placed far from the end of the material, and in non-stationary conditions, i.e., when the strain gauge was placed close enough to the end of the drawn material that the initial estimation based on the drawing force suggested that the length of the material was lower than the expected length of the elastic deformation region. Strain gauges used during analysis measured strains in longitudinal and radial directions.

The last part of the analysis was the Vickers hardness tests performed at the cross-section along the axis of the samples after the drawing process using a TUKON 2500 hardness tester (Buehler, Lake Bluff, IL, USA) with a test load accuracy of +/−1% and an accuracy of the indent’s diameter measurement of 0.02 mm. Starting 0.5 mm from the edge and with the same intervals between measurements, 19 indentations were made on the samples taken from stationary and non-stationary conditions, which allowed for their thorough analysis in comparison with the hardness of the input sample.

## 4. Results and Discussion

### 4.1. Finite Element Method Simulations

**Figure 3:**
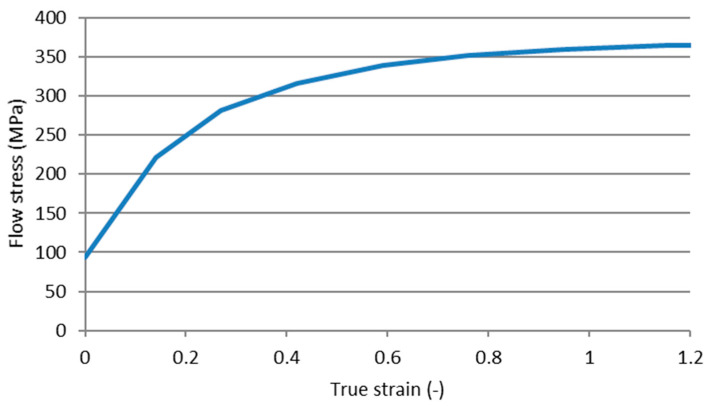
Work hardening curve of Cu-ETP copper in ambient temperature assigned as a model of plastic flow for drawn wire used in FEM (Finite Element Method) simulations.

Numerical analysis of wire drawing process with the use of the FEM method was performed in commercial SFTC DEFORM-3D software (Columbus, OH, USA). The Newton–Raphson method was selected for the analysis with Sparse Solver, which were chosen as an optimal set resulting from previously conducted and verified numerical simulations of drawing processes. A model of the die was set as elastic, and the material properties of tungsten carbide (TC) with 19% of Co were assigned for this object. For the drawn wire, an elastic–plastic model was selected, and material properties of Cu-ETP grade copper were chosen, with a work-hardening curve representing the plastic behavior of the metal. The most important material boundary conditions of FEM simulations are presented in Table 2 and in Figure 3.

The initial diameter of the wire before its deformation was set to 11.55 mm, and a 10 mm diameter was chosen for the die bearing, which gave a total amount of deformation at the level of λ = 1.33 during the process. Figure 3 presents the work hardening values of the representative Cu-ETP grade copper obtained in the uniaxial tensile test of the wire-rod and subsequent samples after each draw up to 11.55, which is approximately at the end of the presented curve (true strain 1.1). The stress value at 0 strain represents the stress for the initial wire rod with a diameter of 20 mm that has not been subjected to the wire drawing process; hence, the 0 strain value has stress of almost 100 MPa, as even the material after annealing is characterized with over a 0 MPa yield strength at 0 strain.

**Figure 4:**
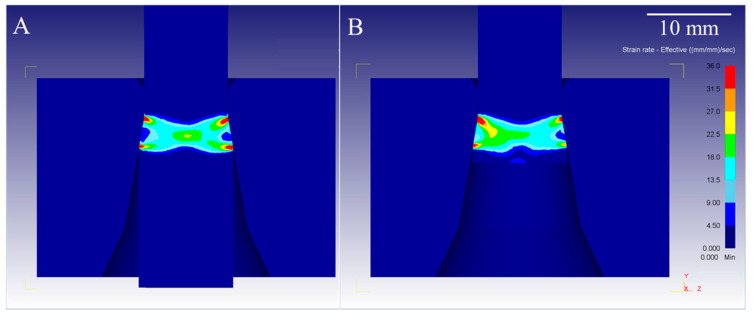
FEM analysis of the strain rate during wire drawing process of copper: (**A**)—stationary conditions; (**B**)—non-stationary conditions.

**Figure 5:**
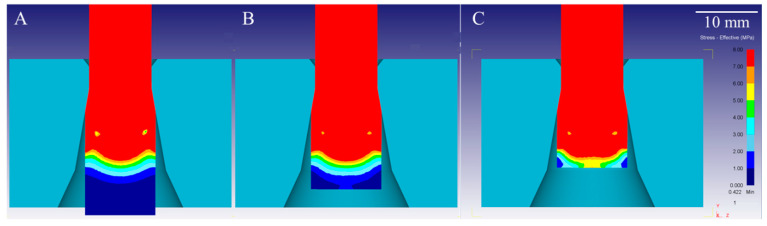
FEM analysis of the stress distribution during wire drawing process of copper: (**A**) stationary conditions; (**B**) non-stationary conditions; (**C**) non-stationary conditions, end of the process.

Results of FEM analysis in the form of effective strain rate characteristics are shown in Figure 4, and stress distribution during the wire drawing process of copper is shown in Figure 5. As can be seen in Figure 4, there is a visible difference in the strain rate of metal, both in the region of wire deformation and directly before the die reduction angle where additional stresses occur. In stationary conditions of the wire drawing process, strain rate values are located mostly close to the surface of the die, with maximum values strain rate exceeding 30 s^−1^. When the process reaches non-stationary conditions, strain rates values increase at the axis of the wire and slightly decrease in the areas close to its surface. As the process proceeds, continuous strain rate distribution changes, and higher values can be observed outside the direct deformation region, close to the end of the wire. The shape of the elastic–plastic deformation region is similar than the aforementioned determined using the method of characteristics described by Pater and Samołyk [11] or obtained by Wistreich in his empirical studies [7]. Comparison of effective stress characteristics in stationary and non-stationary conditions and close to the end of the process also show differences in the stress region before entering the drawing die. In all of the above-mentioned cases, an additional region of elastic stresses was observed inside of the wire before its direct contact with the die reduction angle. The shape of the boundary between the elastic and elastic–plastic deformation regions is lenticular, which is closely related to the analyses conducted by Knych [10]. In order to better visualize the differences in the length of the elastic region, it was set up to show values only up to 8 MPa at 1 MPa intervals. Based on Figure 5B, it may be assessed that the elastic stress length at the surface is shorter (approx. 8.1 mm) than at the axis of the drawn wire (approx. 10.8 mm). Therefore, the shape and length of the elastic deformation region is different than commonly accepted according to the model determined by Perlin [24].

### 4.2. Characterization of the Input Material’s Properties

**Figure 6:**
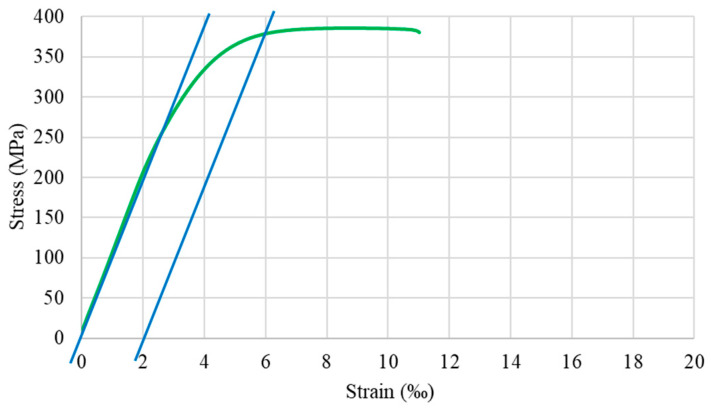
Stress–strain relation of the input material (uniaxial tensile test conducted with the use of strain gauges).

Fundamental mechanical properties of the input material were identified in the uniaxial static tensile tests and Vickers hardness tests. The stress–strain curve obtained in the tensile test with the use of resistance strain gauges enabled us to measure deformations with much higher resolution in comparison with the classic extensometers, and as is easily noticeable, there is a very small range of uniform deformations, which may be estimated at 0.2% or 2‰, as presented in Figure 6. The input material is pre-hardened with approx. strain of 67% with the ultimate tensile strength equal to 388 MPa and similar value of the yield strength. Mean values of both hardness and microhardness measurements conducted with Vickers method were approx. 115 HV, which converted into MPa using the Tabor model, and allowed us to compare the values of hardness and deformation resistance and obtain the proportionality coefficient of 3, being in full consistence with the known literature data for hard materials [43].

After defining the basic mechanical properties of the input material, the elastic constants of the material, i.e., the Poisson’s ratio and the Young’s modulus, were determined. In order to attain these values, ultrasonic, using a supersonic flaw detector OmniScan MX2 (Olympus Corporation, Tokyo, Japan) and strain gauge using Zwick 100kN ProLine (ZwickRoell GmbH & Co. KG, Ulm, Germany) methods were used for comparison, and in both cases similar results were obtained. Table 3 summarizes the properties of the input material used in the experimental tests defined experimentally in our own research.

### 4.3. Analysis of the Temperature Variations during the Process

**Figure 7:**
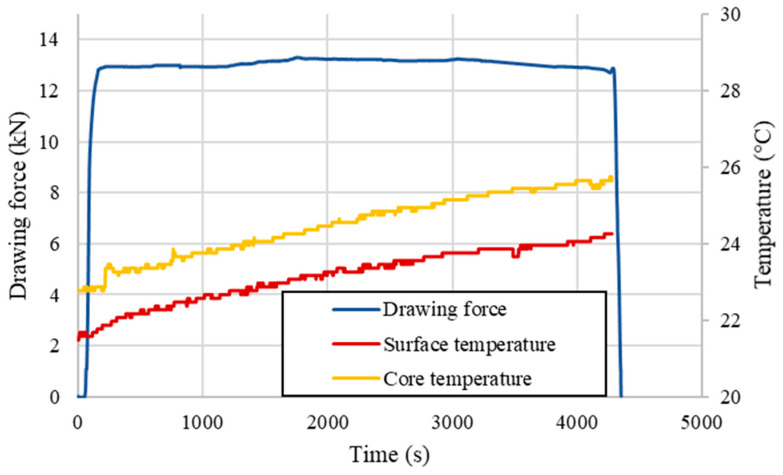
Recorded temperature increase during analyzed wire drawing process (with the use of thermocouples).

**Figure 8:**
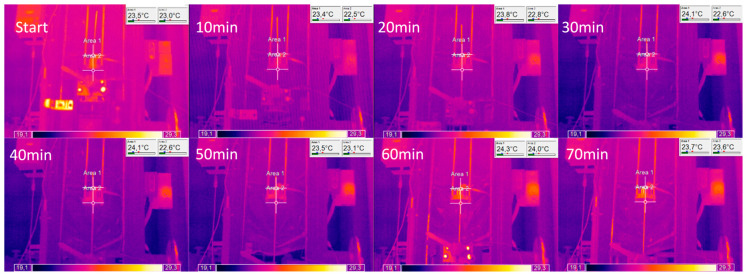
Recorded temperature changes during the analyzed wire drawing process (with the use of thermal imaging camera). The diameter of the die is 10 mm with a 2α die angle of 16° and a bearing length of 1 mm and deformation coefficient of 1.3; material in its initial state is pre-hardened with a true strain of approximately 1.1 (according to Figure 3).

The key research challenge of the current paper was the empirical determination of the length of the elastic deformation region and the values of strains occurring in this region, which would make it possible to verify the aforementioned numerical simulations. In order to perform the experiment, the strain gauge method was used; however, due to the fact that strain gauges show high sensitivity to temperature variations and that the designed test stand excluded the possibility of using compensating sensors into the system, it was necessary to assess the temperature changes of the material throughout the representative drawing process. Such an assessment was made using a flat thermocouple recording the surface temperature of the rod just before entering the die and a second thermocouple placed at the end of the drawn material in a 30 mm deep hole drilled beforehand. The recorded values along with the drawing force are shown in Figure 7. Only a slight increase of the material’s temperature before entering the drawing die of approx. 2 °C was observed, which generated a maximum error of the strain measurement at the level of 0.03‰, which is equal to about 4 MPa (calculated according to Formulas (1) and (2)) and is a fully acceptable value from the point of view of further measurements. Additional analysis of the thermal state of the surface was carried out using a thermal imaging camera throughout the process duration of 70 min, where recorded temperature increased by approx. 1 °C (Figure 8). Since the recorded increase in the process temperature was little to none, the clarity and repeatability of the experiment was undoubtedly confirmed.

(1) $$\varepsilon_{T}=\alpha *\Delta T$$

where $\varepsilon_{T}$—Measurement error; α—Coefficient of thermal expansion; ΔT—Temperature difference

(2) $$\sigma =\varepsilon_{T}*E$$

where *σ*—Accuracy of measurement; *E*—Young’s modulus

### 4.4. Analysis of the Elastic Deformation Region

The main part of the conducted research was the analysis of the shape and length of the elastic deformation region, which was carried out with two initial diameters, the first being 11.55 mm (deformation coefficient λ = 1.33), and the second one after the additional draw being 10.5 mm in diameter (deformation coefficient λ = 1.1). The measured velocity of the material after exiting the drawing die was approx. 0.1 mm/s, which along with the known geometry of the die and the deformation coefficients allowed us to calculate the velocity of the material before entering the die and the mean velocity of the material inside the deformation region. All the above data are presented collectively in Table 4.

**Figure 9:**
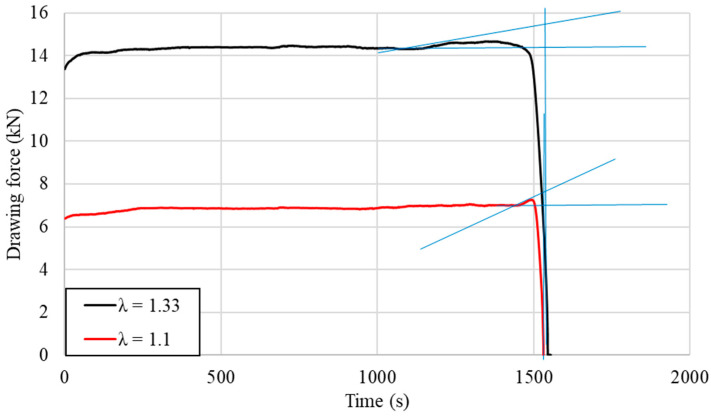
Representative examples of the recorded drawing force of the analyzed process.

In order to verify the hypothesis assumed in the Materials and Methods section, drawing forces for both considered deformation coefficients were measured using five separate samples obtained under the same conditions and of the same length. A detailed analysis of the drawing force values at the end of the process was performed, and in each of the tested cases, an instantaneous increase in the recorded values was observed just before the end of the drawn material. This allowed us to preliminarily estimate the length of the elastic deformation region based on the calculated drawing velocity and the recorded duration of the increase in accordance with Formula (3). Representative examples are presented in Figure 9 along with the marked increases in the drawing force, which are the sole evidence that the stationarity of the process ends, and it becomes non-stationary (it is limited by the blue lines). As is easily noticeable, both the length and the increase of the drawing force are different depending on the applied deformation coefficient.

(3) $$L_{ed}=V_{d^{\prime }}*t_{i}$$

where $L_{ed}$—Estimated length of the elastic deformation region; $V_{d^{\prime }}$—Velocity of the material before entering the drawing die; $t_{i}$—Time of the recorded increase of the drawing force.

Based on the recorded data, calculated velocities of the process for each of the samples and Formula (3), the average values of the length of elastic deformation region for each deformation coefficient were estimated for a given die geometry and are presented in Table 5. It is easy to notice that when considering the samples with deformation coefficient λ equal to 1.33, the length of the elastic deformation region was estimated to be 4 times longer in comparison to the deformation coefficient λ equal to 1.1, which is experimental evidence that the extent of the elastic deformation region is fully related and dependable on the shape of the applied diameter reduction and die geometry, and the estimated differences were quite significant.

**Figure 10:**
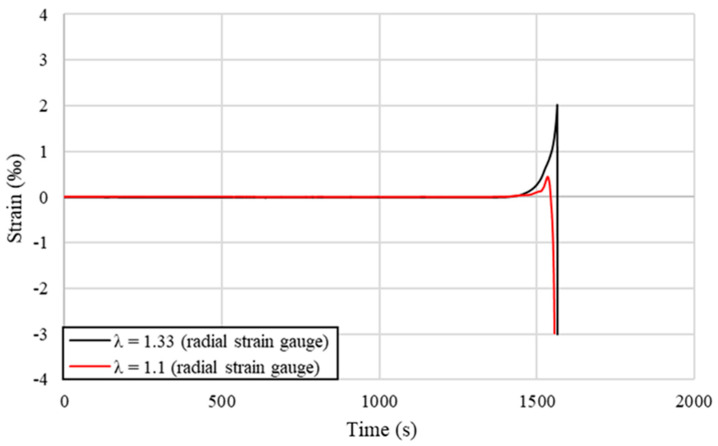
Strain gauge measurements of the elastic deformation in the radial direction (representative examples).

**Figure 11:**
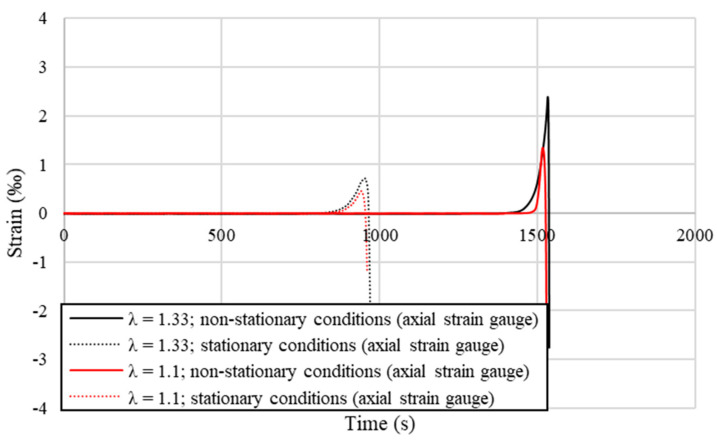
Strain gauge measurements of the elastic deformation in the axial direction (representative examples).

More complex and thorough analysis of the shape and length of the elastic deformation region generated in the material behind the elastic–plastic region was conducted using resistance strain gauges measuring longitudinal and radial strains in stationary and non-stationary conditions, as described in the Materials and Methods section. The graph presented in Figure 10 shows the strains recorded by radial strain gauges placed on the back wall of the input material, while Figure 11 shows the values recorded by the axial strain gauges in both stationary and non-stationary conditions for selected samples. The tests were performed analogically to the preliminary measurements of the drawing force with the same drawing velocity, drawing die geometry and deformation coefficients. In each of the analyzed cases, the recorded values had similar courses; however, the differences may be noticed in the time period, where non-zero values, the maximum strain values, and the rate of its increase were recorded.

From data presented in Figure 10 and Figure 11 it may be concluded that recorded strains take much higher values when the deformation coefficient is increased, which is especially visible in terms of radial strains presented in Figure 10, where the values peak at the value 4 times higher when the deformation coefficient λ is increased from 1.1 to 1.33. This is not as evident in stationary conditions in terms of the axial strains presented in Figure 11. However, it may be stated that the length of the recorded strain and consequently the length of the elastic deformation region in stationary conditions is longer, regardless of the applied deformation coefficient, than in non-stationary conditions, even though the growth rate of the strain as it approaches the dies and the maximum recorded value of strain is much smaller. Taking that into account, it might be an experimental confirmation of the observed in FEM simulation differences in the length of the elastic deformation region near the wire surface and at the wire axis, depending on the process conditions.

The recorded data shown in Figure 10 and Figure 11 are collectively presented in Table 6 along with the length of the elastic deformation region calculated based on Formula (3). As might be easily noticed in the case of the lower deformation coefficient λ = 1.1, these values were similar to those preliminarily estimated on the basis of the drawing force measurements; however, considering the higher deformation coefficient λ = 1.33, it is noticeable that these values are more than two times lower. Calculated results confirm the previously observed relation stating that the length of the elastic deformation region is shorter when the applied deformation coefficient is lower. Maximum recorded values were sometimes even a few times higher when the diameter reduction was higher. What is worth noting is that the length of the strain recorded in the radial direction is longer than in the axial direction in non-stationary conditions, and in addition the strain gauge is placed further on the longitudinal section of the tested samples. This means that for a given cross-section of the material, firstly the elastic deformation appears on the radial strain gauge and slightly later on the axial strain gauge, which also confirms experimentally the shape of the elastic deformation region observed in the FEM simulation visible in Figure 5.

**Figure 12:**
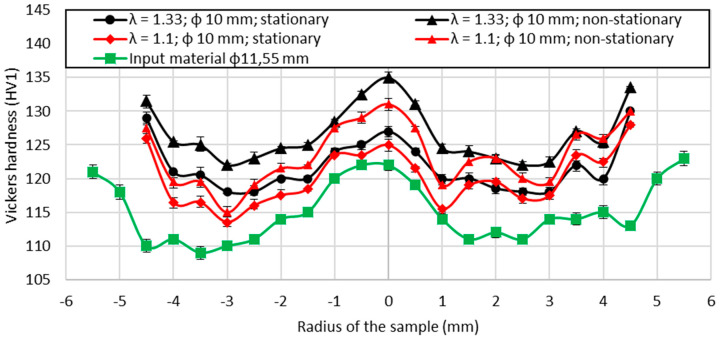
Vickers hardness measured at the axis of the cross-section (average values).

**Figure 13:**
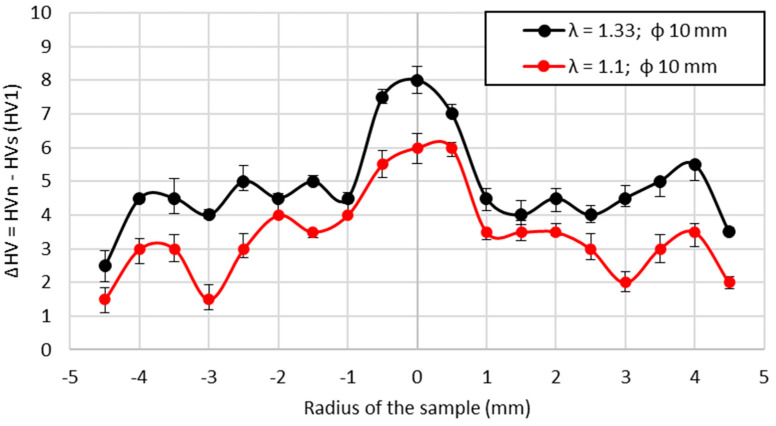
The difference in hardness measurements of the cross-section in non-stationary conditions (HVn) and stationary conditions (HVs).

The final stage of the carried-out research included Vickers hardness tests conducted on the cross-section of the samples with a 10 mm diameter after the drawing process. Samples of the cross-section were taken from stationary and non-stationary phases of the process for both applied deformation coefficients. The average values calculated from all 5 drawn samples are presented in Figure 12 along with the marked error bars. The analysis of the results included the calculation of the difference between the mean values of non-stationary and stationary conditions, which is presented in Figure 13, again along with the marked error bars. It may be noticed that in the axis of the material, the hardness values in non-stationary conditions are much higher, which indicates greater deformation in this area. Slightly lower values were observed at the surface of the drawn material, and definitely the lowest measured values were recorded between the axis and the edge of the material. However, in stationary conditions, the calculated hardness values were lower at the axis than at the surface of the wire. The calculated differences clearly show that the greatest differences between stationary and non-stationary conditions in the drawing process occur in the axis of the material. The measured values and the calculated differences are greater for samples taken from the materials subjected to higher deformation coefficient. Considering the hardness of the input material there was also a visible variation in recorded values along the axis of the sample which might be a consequence of the heterogeneity of deformation along the cross-section. On the basis of conducted research, it might be concluded that the highest local strains are localized at the surface and at the axis, which is similar to the FEM simulation results known from the literature [12,44]. Such observation is closely related to the location of the beginning of the elastic and elastic–plastic deformation region in both stationary and non-stationary conditions according to FEM simulations, i.e., the boundary of these regions did not change significantly at the surface, while the change is clearly noticeable at the axis of the wire.

## 5. Conclusions

Taking into consideration the obtained FEM simulations, empirical research results and conducted calculations, the following may be stated:

(1) The results of the FEM simulation indicate the differentiation of the shape of the elastic deformation region in stationary conditions, where a much greater range of deformations was recorded at the wire surface in comparison with non-stationary conditions, i.e., at the end of the process, where the values of elastic deformations are greater along the material axis.
(2) The presence of the elastic deformation region was indirectly verified by drawing force measurements with different drawing parameters (various deformation coefficients λ). The increase in the recorded drawing force at the end of the process was related to the potential length of the elastic deformation region. It was proven that with the increase of the deformation coefficient, the length of this region increases even several times and can reach even 28.6 mm in length.
(3) It was proven that the range of the elastic deformation region in non-stationary conditions is greater along the wire axis than at the surface using strain gauge method. In addition, it was stated that the length of the elastic deformation region at the surface of the drawn wire in stationary conditions is definitely greater than at the material surface in non-stationary conditions. The tests confirmed that the applied deformation coefficient affects the lengths and shape of the considered strain regions.
(4) The hardness tests of samples taken from the cross-section in non-stationary phase of the process show that the recorded values at the axis were the highest with slightly lower values at the surface of the material and definitely the lowest in between. However, considering the samples taken from stationary conditions the measured values were higher at the edge of the material than at the axis, which confirms the differences in the amount of strain depending on the process phase. The greatest differences between the stationary and non-stationary conditions are visible at the axis of the drawn material. The measured values are again influenced by the applied deformation coefficient throughout the drawing process.
(5) The shape of the elastic deformation region before entering the die reduction angle was confirmed to be in compliance with the FEM simulations conducted throughout this research paper among the tested areas.

## Figures and Tables

**Table 1:** Chemical composition of the input material (ETP grade copper (electrolytic tough pitch)).

Cu	Ag	Bi	Pb	Sb	Sn	Zn	S	O	Other
(wt %)									
99.97	0.00128	0.00002	0.00012	0.00003	0.00003	0.00003	0.0052	0.02	Rest

**Table 2:** Set of most important material boundary conditions assigned for the numerical simulation of wire drawing.

Group	Parameter	Wire—Cu-ETP	Die—TC
Material properties	Young modulus (GPa)	119	496
	Poisson’s ratio (-)	0.35	0.24
	Model of plastic flow	$\sigma_{p}=\sigma_{p}\left(\varepsilon \right)$	-
Other boundary conditions	Model of friction	Coulomb	
	Friction coefficient	µ = 0.07	
	Initial true strain before drawing	1.1	
Discretization	Type of mesh	Tetrahedrons	Tetrahedrons
	Number of elements	50,000	100,000

**Table 3:** Mechanical and elastic properties of the input material.

Parameter	Input Material φ 11.55 mm
Young’s modulus (ultrasonic) (GPa)	118
Young’s modulus (strain gauges) (GPa)	119
Poisson’s ratio (ultrasonic) (-)	0.36
Poisson’s ratio (strain gauges) (-)	0.35
Ultimate Tensile Strength (MPa)	388
Yield Strength (MPa)	385
Vickers hardness (HV1)	115.2
Vickers microhardness (HV0.3)	115.9

**Table 4:** Drawing die dimensions along with the deformation coefficient and velocity of the analyzed wire drawing process.

Parameter	Value	
Diameter (mm)	10	
2α die angle (°)	16	
Bearing length (mm)	1	
Deformation coefficient (λ) (-)	1.1	1.33
Calculated velocity of the material before entering the drawing die (mm/s)	0.0928	0.0767
Calculated velocity of the material inside the deformation region (mm/s)	0.0976	0.0895
Measured velocity of the material after exiting the drawing die (mm/s)	0.1023	

**Table 5:** Estimated lengths of the elastic deformation region based on the measured drawing force (average values) during the wire drawing process with the velocity of 0.1 mm/s and deformation coefficients of 1.1 and 1.33.

Parameter	Value	
Deformation coefficient (λ) (-)	1.1	1.33
Average drawing force (kN)	6.45	14.39
Recorded time of the drawing force increase (s)	76	373
Estimated length of the elastic region (mm)	7.1	28.6

**Table 6:** Estimated lengths of the elastic deformation region-based strain gauge measurements (average values) during the wire drawing process with a velocity of 0.1 mm/s and deformation coefficients of 1.1 and 1.33.

Parameter	Value			
Deformation coefficient (λ) (-)	1.1		1.33	
*Strain gauge in the radial direction*				
Recorded time of the drawing force increase (s)	91		140	
Estimated length of the elastic region (mm)	8.44		10.74	
Recorded strain value (‰)	0.45		2.02	
*Strain gauge in the axial direction*				
Conditions of the measurement (stationary (S)/non-stationary (N))	S	N	S	N
Recorded time of the drawing force increase (s)	100	49	152	119
Estimated length of the elastic region (mm)	9.28	4.55	11.66	9.13
Recorded maximum strain value (‰)	0.42	1.33	0.71	2.36

## Data Availability

Data available upon request.
